# Sudden Death of a Young Man Due to Massive Haemoptysis Associated With Pulmonary Tuberculosis: A Rare Case

**DOI:** 10.7759/cureus.47635

**Published:** 2023-10-25

**Authors:** Matteo Antonio Sacco, Saverio Gualtieri, Maria Cristina Verrina, Pietrantonio Ricci, Isabella Aquila

**Affiliations:** 1 Department of Medical and Surgical Sciences, Magna Graecia University of Catanzaro, Catanzaro, ITA

**Keywords:** hemoptysis, infection, asphyxia, autopsy, tuberculosis

## Abstract

Asphyxia includes a large number of possible causes and mechanisms, such as infectious diseases of the lungs. Among these, tuberculosis (TB) is one of the main infectious causes of death worldwide. The burden of TB is very high, by virtue of antibiotic resistance and the risk of other concomitant infections, such as HIV. A major problem is related to the omitted diagnosis of TB infection which, in some cases, may remain completely unknown to the patient, with a significant increase in mortality and morbidity risk. There has been a decrease in the reported cases of sudden death due to TB, especially that associated with hemoptysis and aspiration of blood. In this report, we describe a case of sudden death that occurred in a young male in the workplace, under unclear circumstances. The autopsy detected acute asphyxia due to a massive pulmonary hemorrhage in the individual, who was apparently suffering from TB. The autopsy proved to be essential for understanding the cause of death and investigating the adequacy of the health surveillance measures carried out on the individual.

## Introduction

Classification of asphyxia

Asphyxia is a condition that occurs when the body receives insufficient oxygen, causing the organs and tissues to be inadequately oxygenated. Asphyxia fatalities can arise from obstructions to the respiratory tract that prevent air from entering or the compression of nerves and vessels in the neck. The physical components of asphyxia are responsible for defining various manners, such as suffocation, smothering, hanging, choking, and death caused by the inhalation of foreign objects. Pulmonary infections, such as tuberculosis (TB), can rarely cause asphyxiation [1]. In this type of asphyxia, the victim experiences suffocation due to severe pulmonary bronchial or intra-alveolar hemorrhage with massive aspiration of blood. 

Epidemiology of TB

From an epidemiological point of view, the global health crisis that is TB cannot be ignored. The World Health Organization (WHO) reports that one-third of the world's population, approximately two billion people, have been exposed to the *Mycobacterium tuberculosis* pathogen [2,3]. This disease has become one of the top 10 causes of death worldwide and is the primary cause of death among HIV-positive individuals [4]. TB is a major global health issue, with an estimated 1.6 million deaths in 2021 [5]. The risk of developing TB is higher in the first 12-18 months following the infection, and several medical conditions such as HIV infection, silicosis and exposure to silica dust, malnutrition and protein imbalance, and treatment with immunosuppressive drugs [6], can increase the risk of TB. It is particularly severe among people with HIV and is a leading cause of death from an infectious agent [3]. 

Identification of TB

The emergence of multidrug-resistant and extensively drug-resistant TB has increased the urgency to address TB on a global level [7]. Despite effective antibiotic therapies, many cases of TB go unnoticed, particularly in instances where the infection is latent or when the patient lacks access to treatment [8]. Identifying the early signs and symptoms of TB is of utmost importance, as it can ensure that the infected individuals receive proper treatment and prevent the spread of the disease. If left untreated, TB can lead to complications with a mortality rate exceeding 50% [9]. The literature has documented rare cases of sudden death resulting from TB-related complications such as pericarditis, pneumothorax, myocarditis, and coronary arteritis [6,10-12]. In these cases, the patient had not received treatment, and TB was only discovered post-mortem during an autopsy.

## Case presentation

Scene findings

A 22-year-old refugee, coming from a high prevalence geographical area (Ivory Coast), was discovered deceased in a camp during work, with evidence of bleeding from the nose and mouth. The body was found near a tree. Authorities initially suspected a traumatic death due to the presence of blood on the scene. For this reason, an autopsy was requested.

Autopsy findings

**Figure 1:**
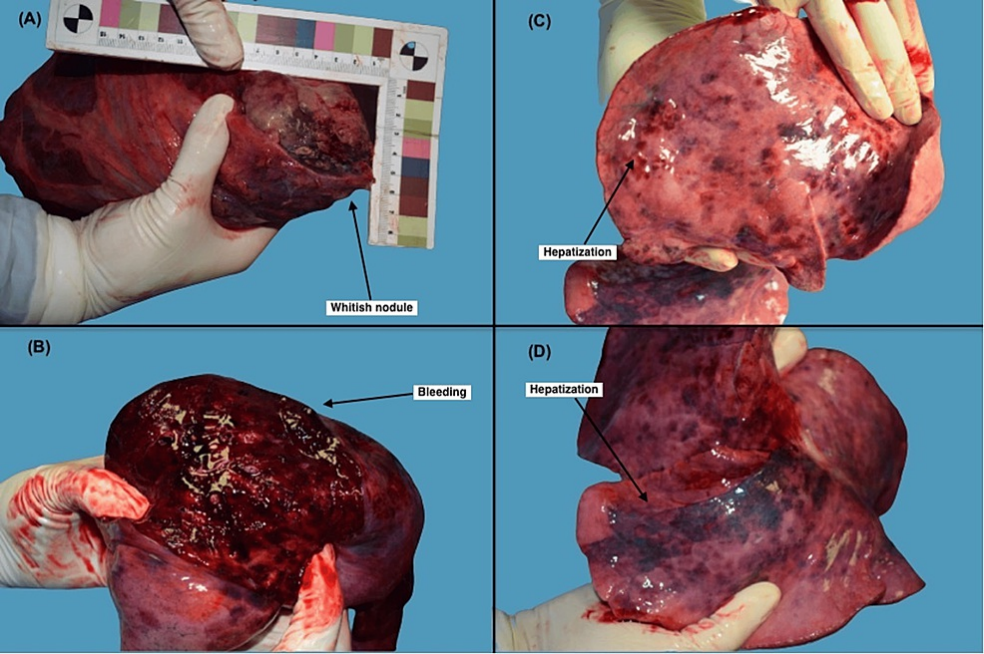
Autopsy findings show lungs displayed a significant infection marked by hepatization, diffuse bleeding areas, fibrosis, and a whitish nodule on the left lung with surrounding necrosis

The external body examination did not show signs of a struggle. At autopsy, the lungs displayed a significant infection marked by hepatization, diffuse bleeding areas, fibrosis, and a whitish nodule on the left lung with surrounding necrosis (Figure 1). Upon sectioning, the lungs exhibited widespread hemorrhage. Additionally, a large amount of red blood was present in the opening of the stomach, and blood was also found in the trachea because it was inhaled. Histological examination uncovered bronchopneumonia foci with giant cells, as well as hemorrhage in the alveoli. Corresponding with the whitish nodule in the left pulmonary apex were foci of necrosis and inflammation, i.e. Langhans cells, as pathognomonic signs of TB. The autopsy evidenced that death resulted from respiratory failure due to pulmonary hemorrhage with massive aspiration of blood as a complication of TB.

## Discussion

**Figure 2:**
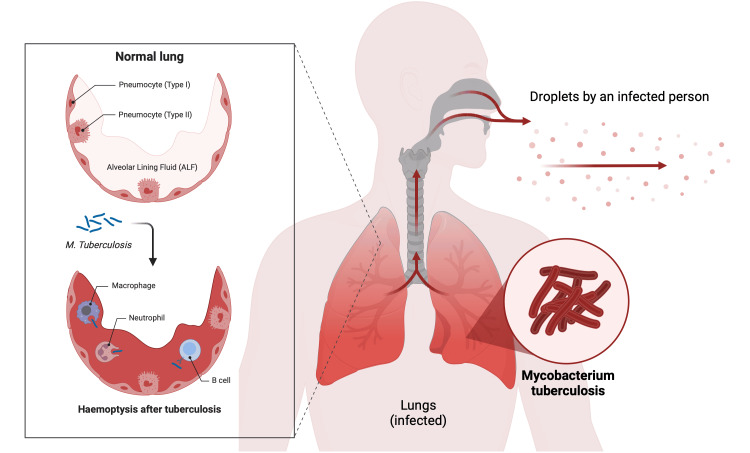
Physiopathology of hemoptysis in Mycobacterium tuberculosis infection Image created with Biorender.com

Pulmonary hemorrhage is a serious complication that can lead to tracheobronchial obstruction and ventilatory failure. It can have various causes, including autoimmune systemic vasculitis, pulmonary infection, fungal infection, lung cancer, lung abscess, and coagulopathy, and thus requires careful differential diagnosis (Figure 2) [13-17]. This condition is characterized by damage to small blood vessels in the lung tissue leading to bleeding.

One of the deadliest complications that can occur with TB is massive hemoptysis, the coughing up of blood from the lower respiratory tract. Hemoptysis is a common symptom of pulmonary hemorrhage, which is mostly caused by damage to the bronchial arteries with higher pressure than the pulmonary arteries, resulting in massive hemorrhage. Diagnosis and treatment of pulmonary hemorrhage are difficult and complex, requiring comprehensive evaluation and adequate therapy. Rapid and accurate detection of this complication by radiography is critical, most appropriately using bronchial artery embolization (BAE). According to the literature, BAE is an effective and safe treatment option for the treatment of hemoptysis in TB patients [2,3,6,11,18]. From a forensic point of view, the hemorrhage bleeding, with hemoptysis, may mimic severe injury, and the death scene might look suspicious for a possible accident or crime since there is usually a lot of blood.

Autopsy can identify previously undetected cases of TB. Autopsy findings associated with TB deaths have been extensively studied showing that the most common causes include bronchopneumonia, pleural effusions, and miliary TB [19-21]. Pulmonary pathology in patients with autopsy-confirmed pulmonary TB is often accompanied by bacterial pneumonia, pneumocystis pneumonia, cytomegalovirus pneumonia, pulmonary thromboembolic disease, interstitial lung disease, and bronchiolitis obliterans pneumonia. Acid-fast bacteria have been found in the lungs of most cadavers with TB and TB bronchopneumonia is the most common finding [1]. Microscopic examination of organs showed Langhans giant cells, noncaseating epithelioid granulomas, and Ziehl-Nielsen stained acid-fast bacilli as the most common findings.

Complications of TB leading to sudden death are rare and few cases have been reported [19-21]. The current case demonstrates the potential for abrupt fatality due to TB-related complications, even among very young individuals who are unaware of their infection and have not received any treatment. In this case, autopsy was fundamental because it identified an unknown and untreated TB. The case shows the importance of evaluating an autopsy performed in cases of unexpected death, particularly among young people, to investigate the cause of death and solve doubts. This is especially important when death occurs in occupational settings, where it is imperative to verify all medical preventive measures of the employee for legal purposes, as in the case reported [21,22].

## Conclusions

The causes of sudden death in young people can vary. For this reason, the etiology of sudden and unexplained death in similar cases should be always investigated, especially when it occurs in workplaces. The role of unknown infectious diseases, such as TB, in the genesis of these fatal events should not be underestimated. These events may include acute episodes of asphyxiation, as in the case reported. In these cases of sudden and unexplained death, the autopsy represents an essential assessment to discover the cause of death and it should include toxicological investigations, in order to assess any intoxication. Autopsy is mandatory in similar cases that occur in workplaces to evaluate the application of hygiene and safety measures at work with appropriate medical examinations of workers. Preventive measures should include accurate and periodic medical visits, monitoring through laboratory investigations, controls of vaccination, and investigation of any infectious diseases or other comorbidities.
